# The Effect of Eucalyptol on Nursing Home Residents

**DOI:** 10.1038/s41598-020-61045-8

**Published:** 2020-03-04

**Authors:** Seiko Goto, Hinako Suzuki, Toshinori Nakagawa, Kuniyoshi Shimizu

**Affiliations:** 1 Nagasaki University, School of Environmental Science, Nagasaki, 852-8521 Japan; 2 Shiga University, School of Environmental Science, Shiga, 522-8533 Japan; 3 Kyushu University, Faculty of Agriculture, Fukuoka, 819-0395 Japan

**Keywords:** Environmental impact, Geriatrics

## Abstract

Eucalyptol is one of the most popular volatile components. It is used in many essential oils for relieving sinus and lung congestion caused by a variety of conditions. This pilot study sought to analyze clinical evidence for the effect of the scent of eucalyptol on the cognitive function of elderly people. Seventy nursing-home residents with cognitive impairment were recruited. Three one-week experiments were performed: eucalyptol scent was diffused in bedrooms with a diffuser only at wake-up time in the first experiment, and at wake-up time and bedtime in the second and third experiments. Results showed that although an improvement was not seen when using Mini Mental State Examination (MMSE) and Cohen-Mansfild Agitation Inventory (CMAI) measures, Dementia Behavior Disturbance Scale (DBD) scores improved significantly, even though no subject reported perceiving the scent. The significant improvements of the behaviour were found not only among the subjects whose room had a diffuser but also among the subjects who were exposed to an unperceivable level of eucalyptol drifted in the living room.

## Introduction

Aromatherapy with essential oils has been used in many countries from ancient times. Aromas trigger physical and mood improvement via the olfactory system^1,2^. Inhaled air containing a scent can reach the circulatory system but can also stimulate brain areas directly via receptors in the olfactory epithelium. Once the signals reach the olfactory cortex, release of neurotransmitters and neuropeptides takes place, which results in a marked effect on emotions^3–5^. Previous studies by Goto showed that visiting a small indoor garden with 20 pots of chrysanthemums for 15 minutes improved the mood of elderly subjects with dementia^6^. Because subjects became more alert and their mood significantly improved while observing the garden, the effects of visiting the garden were hypothesized to involve olfactory stimulation^7,8^.

Pursuing this finding is not straightforward as different parts of plants emit different scents, and scents from the same parts can differ based on freshness. Even within the same plant family, different varieties can have very different volatile components^9^. Because the volatile components of plants are uncertain, the specific volatile organic compounds in commercial essential oils bearing the same plant name, i.e. lavender or rose, differ by brand. Furthermore, although essential oils are composed of multiple volatile components, there has been little in-depth research on the efficacy of a single component. Although inhalation of essential oils has been traditionally practiced as aromatherapy, the concentrations, dosage, duration, and frequency of usage are not standardized in practice^10^.

Because the mode of administration of an essential oil and its content, as well as the effects of each volatile, are not clear, most studies of aromatherapy is of scientifically inadequate quality^11^. Even if the scent is usually effective, using different essential oils with different volatile components at different concentrations and frequencies may have the either positive or negative effects. It is important, therefore, to identify the effects of a single volatile with controlled dosage and duration, particularly if the scent is to be used for a frail population, such as elderly people with dementia.

In the current study we focused on eucalyptol, one of the main volatiles of chrysanthemums^12,13^. Eucalyptol (1,8-cineole) is a monoterpenoid compound present in large amounts in a variety of plants often used in cosmetics^14,15^. It has been used as an ingredient in many medicinal products to treat bronchitis, sinusitis, and chronic rhinitis, and also asthma, and rhinosinusitis^16,17^. Eucalyptol also enhances blood circulation^18^, and Ambrosch reported that dermal application of eucalyptol increased brain activation in the frontal cortex and significantly improved brain function related to working memory in rats^19^. Based on this evidence, we measured the effects of eucalyptol scent on elderly Japanese adults with dementia who were residents in a nursing home^20^.

## Methods

### The site

**Figure 1:**
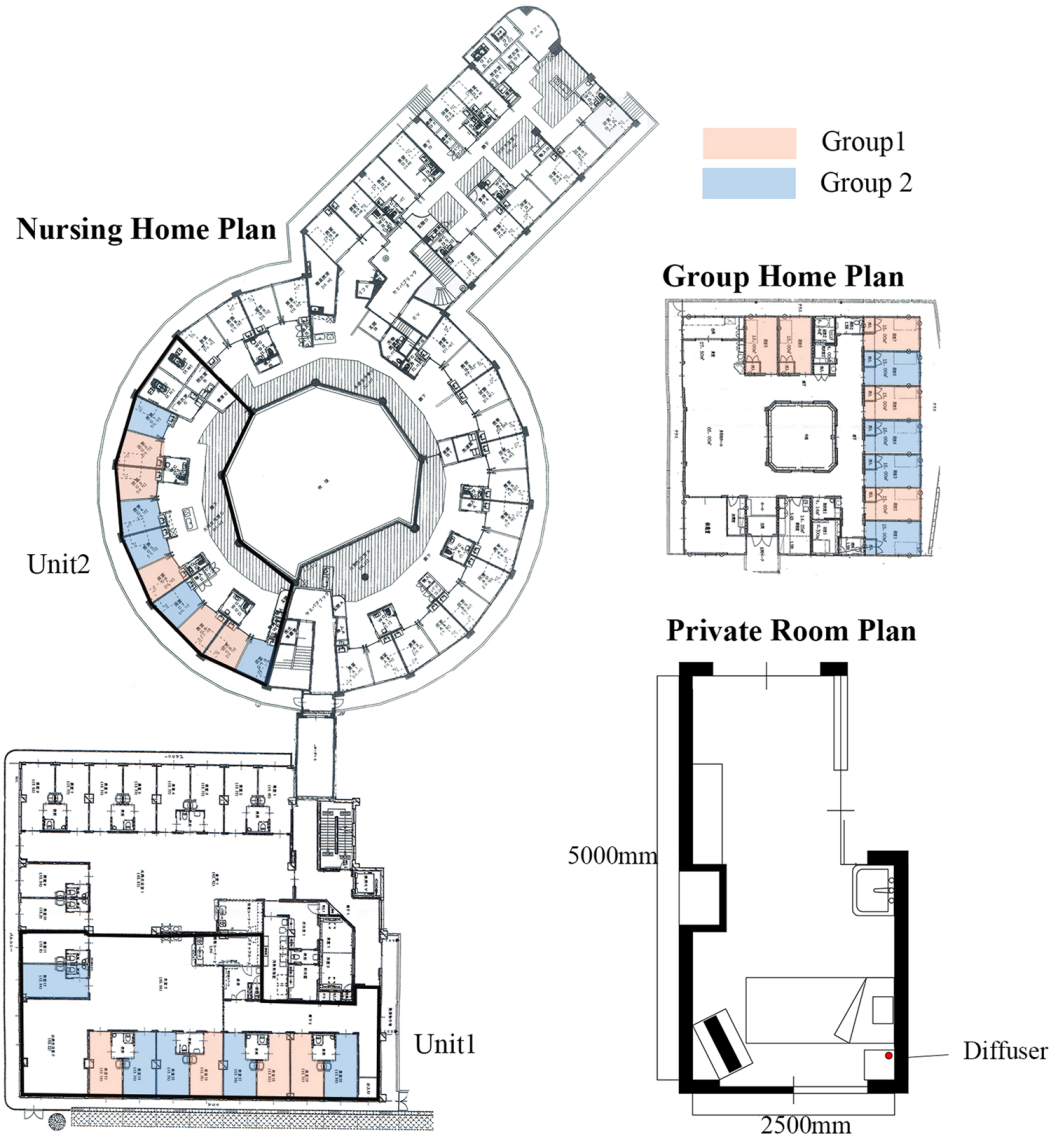
Floor Plan of the group home and a typical room plan. Pink indicates the room with a diffuser. Blue indicates the room with no diffuser.

The experimental site was located in Nozomi-no-mori (N. Home) in Nagasaki, Japan. N. Home is a facility providing daycare as well as short- and long-term stays for elders needing care. The long-term stay facility is comprised of a group home for elders with mild disabilities and a nursing home for those with serious disabilities. There are 9 single rooms in the group home, and 5 units of 10 single rooms in the nursing home. (Fig. 1) Residents are assigned to either the group home or nursing home based on their Care Need Level, a level assessed by seven scales established by the Ministry of Health^21^.

N. home assigns people from Care Need Level 1 and 2 to the group home, and people with Care Need Levels 3 and above to the nursing home. Seven to ten staff members are assigned to each unit for each of 3 work shifts. The policy of N. Home is to care for residents according to their personal lifestyle. Therefore, N. Home does not set times for waking, eating, bathing, toileting, and going to bed. They serve meals when residents desire them. Although they provide cultural activities such as crafts, calligraphy, and singing in each unit twice a week, they do not force residents to participate. Since the nursing home does not have a medical doctor, it does not provide medication. Residents either visit the nearby hospital or call a doctor if necessary.

The group home is composed of 9 single rooms (5 × 3 m) with a common kitchen and living space. The nursing home is composed of 5 units of 10 (4 × 3 m) single rooms with a common living room. Each room has a bed, side table, closet, and chest, and the layout of the rooms is identical. The temperature is controlled by air conditioners installed on the ceiling of each room, and the windows are closed most of the day. The air conditioners were run occasionally during the period of the experiment if the room temperature exceeded 22 + 1. Figure 1 shows the floor plans of the group home (Group Home and Control 2) and the nursing home (unit 1, unit 2, and Control 1). Pink indicates subjects’ rooms that were supplied with a diffuser, blue indicates subjects’ rooms with no diffuser, and green indicates the subject’s rooms in with no diffuser anywhere within the unit.

### Study design

Three one-week single blind experiments (6/18–24/2018, 10/12–18/2018, 11/15–21/2019) were conducted in the nursing home. Before and after each test, subjects and controls were tested using the MMSE (Mini-Mental State Examination)^22^, DBD (Dementia Behavior Disturbance scale)^23^, and CMAI (Cohen-Mansfield Agitation Inventory)^24^.

In the first experiment, 8 drops of eucalyptol oil were dispersed without dilution using an electric diffuser (Truffle, Tree of Life Company, Japan) set on a side table approximately 50 cm away from the subject’s pillow, and started one hour before the subject’s wake time. In the second and the third experiments, the same amount of eucalyptol was diffused in the same setting for one hour before the subject’s wake time and also for 60 minutes at bedtime. Except for diffusing the scent in the subjects’ rooms, no change was made in their daily routine.

### Subjects

Experiments were performed in accordance with relevant guidelines and regulations of Nagasaki University with the agreement of N. Home. Human research protocols for the study were approved by the Ethics Committee of Nagasaki University. The Inclusion Criteria for Study Selection were residents of N. Home. Exclusion criteria for study selection were insufficient health stability to be able to live in the unit during the entire study period. Seventy subjects were recruited for the experiment from N. Home residents. For the 1^st^ and 2^nd^ experiments, family members of 14 subjects gave written informed consent to have a diffuser in their bedrooms (Group 1), and for 13 subjects not to have a diffuser in their rooms (Group 2) from Unit 1, Unit 2, and the Group Home. Many residents of units changed within one year in N. Home. For the 3^rd^ experiment, 13 subjects who would have a diffuser in their bedrooms (Group 1), and 14 subjects who would not have a diffuser (Group 2) were recruited from Unit 1, Unit 2, and the Group Home. In addition, 16 subjects were recruited from units without a diffuser (Group 3) from Control 1 and Control 2, and with written informed consent obtained from a family member.

**Figure 2:**
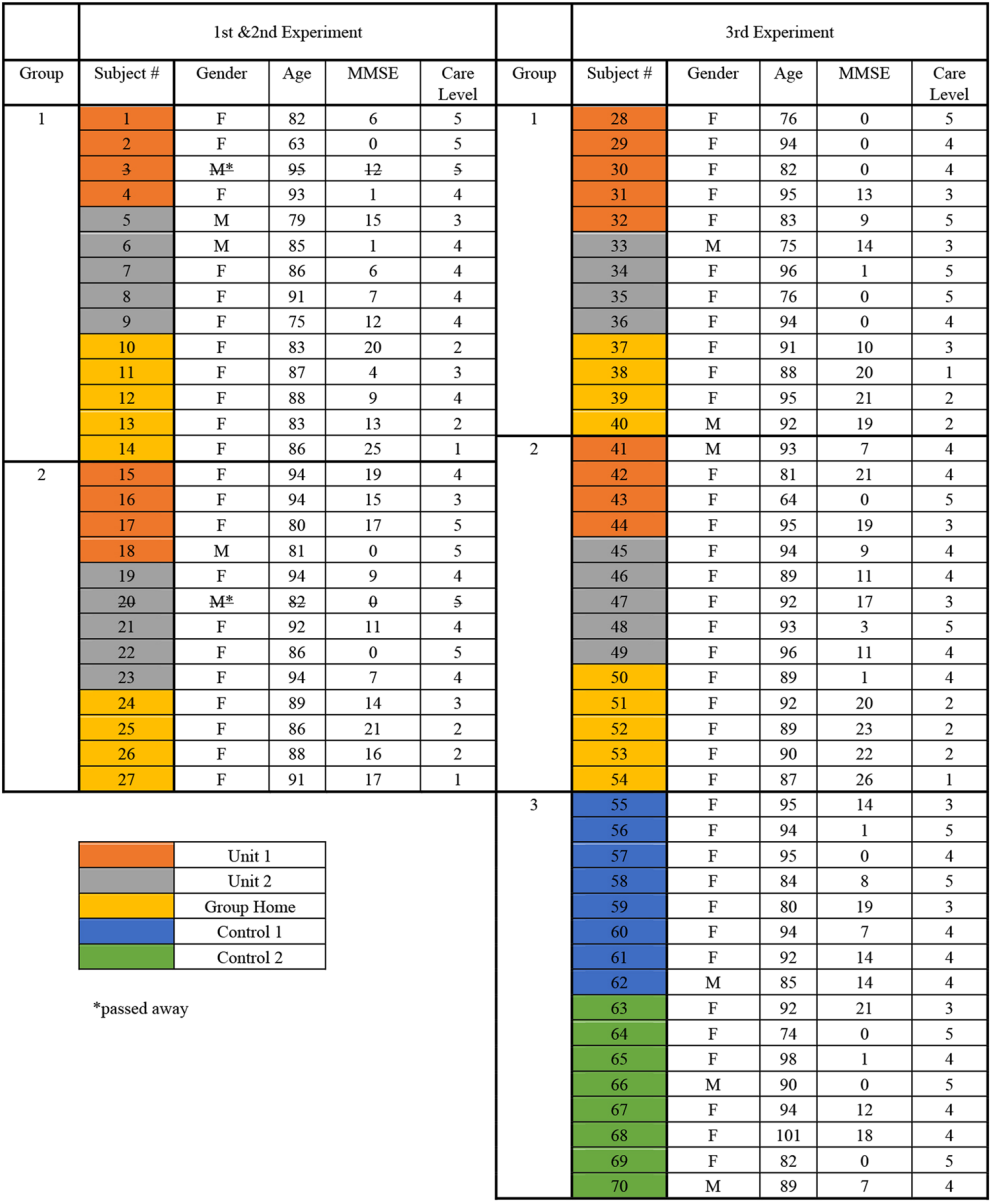
Subjects’ information (gender, age, MMSE, Care Level).

There were 11 females (average age 83.3 + 8.2) and 3 males (average age 86.3 + 8.0) for Group 1, and 11 females (average age 89.8 + 4.5) and 2 males (average age 81.5 + 0.7) for Group 2 in the 1st and 2nd experiments. There were 11 females (average age 88.1 + 7.3) and 2 males (average age 83.5 + 8.5) for Group 1, 13 females (average age 88.5 + 8.0) and 1 male (93 years old), and 13 females (average age 90.3 + 7.5) and 3 males (average age 88.0 + 2.1) for Group 3 in the 3rd experiment. The MMSE, DBD, and CMAI values before experiments were not significantly different between groups. No participants were taking any medication or seeing doctors during the period of experiments. All residents followed their routine schedules in their unit, and no subjects dropped out, or participated in a new activity during the experiment. During the two one-week experiments, two subjects died – one from Group 1 and one from Group 2 – between the 1^st^ and 2^nd^ experiment. Detailed information for each subject is provided in figure 2.

### Questionnaire

Before and after the two experiments, all subjects were tested for their level of cognitive impairment and behavioral symptoms using MMSE, DBD and CMAI. The MMSE (Mini–Mental State Examination) is a 30-point questionnaire to measure cognitive impairment. The test takes between 5 and 10 minutes and examines functions such as attention, calculation, recall, language, ability to follow simple commands, and orientation. DBD (Dementia Behavior Disturbance Scale) is a questionnaire for caregivers to measure the behavioral symptoms of patients. The DBD scale is comprised of 28 questions about dementia-related behavior, scored by an observer on a 5-point scale (Score 0 = Never; 4 = Have the symptom all the time). The CMAI (Cohen-Mansfield Agitation Inventory) is a questionnaire for caregivers to assess the agitation that frequently accompanies cognitive impairment. The questionnaire consists of 14 questions relating to aggressive behaviors and 15 relating to non-aggressive behaviors on a 7-point scale. (Supplementary Document) The DBD and CMAI questionnaires were filled out by the chief caregiver in charge of the unit without notifying of the presence or absence of a diffuser in the subjects’ room.

Nonparametric tests (Wilcoxon test and Friedman test, SPSS software) were applied to determine the significant of the differences due to the small sample size of each group. Significance was established at *p* < 0. 05: *and a result was considered to be trending at *p* < 0. 1. For the analysis of change in MMSE score, subjects whose initial MMSE = 0 were excluded. To assess the interaction among dependent variables, stepwise regression methods were performed for the change in MMSE, CMAI, and DBD using the subject or control, subjects’ age, gender, and the Level of Care Level as dependent variables.

In addition to MMSE, DBD, and CMAI questionnaire, the caregivers of the morning shift were asked to fill out one question every day. Because the staff member changes every day, it was impossible to ask questions based on continuous observation of residents in N. Home. However, the caregivers of the morning shift were asked to the subjects who had the diffuser in the room if they perceive any smell. This question was made every day when the caregiver helps the subject to wake up in his/her room.

### The diffuser and verification methods for the scent

Eucalyptol was diffused by a Truffle diffuser, which is a type of nebulizing diffuser that does not use a water diluent and thus produces a potent mist. It operates on a 2-minute-on and 1-minute-off interval cycle for 2 hours. One Truffle is designed to fill up to a 32 m^2^ room with scent in 2 minutes. The temperature of the experimental rooms was maintained at 22 ± 1 °C. The diffuser was located under the side table next to the bed, 50 cm away from the pillow, where it was invisible from the bed. (Fig. 1) After the diffuser had been operating for 10 minutes, the scent was noticeable to the experimenters in the room. Although the scent of eucalyptol was noticeable to all caregivers and staff members, no subject noticed according to the record of the subjects’ response to the caregivers.

The level of eucalyptol was calculated by averaging the results of 3 air samples collected for 1 minute using a TENAX TA tube (Tenax TA; Gerstel GmbH & Co.KG). The air in the bedroom was collected for 1 minute with a flow rate of the pump 0.05ℓ/min after 15 minutes of operation of the diffuser. The air outside the bedroom was collected for 1 minute with a flow rate 0.15ℓ/min at the three locations.

The tubes with volatiles were thermally desorbed to a gas chromatography-mass spectrometry system (GC/MS; Agilent 7890 A/5975 C) with a thermal desorption unit (TDU; Gerstel GmbH & Co.KG). The GC/MS was equipped with a DB-5MS column (30 m × 0.25 mm; 0.25-μm film thickness; Agilent Technologies). Cryo-injection (from −100 to 40 °C in 15 min) allowed volatiles to enter the injection port of the GC/MS system. The oven temperature program was as follows: 40 °C hold for 5 min, then increased to 85 °C at a rate of 5 °C/min, and then increased to 230 °C at a rate of 20 °C/min. Helium was used as the carrier gas at a flow rate of 1 ml/min. For quantitative analysis, 1 μl of a benzaldehyde solution (200 µl/l, acetone) was added as the internal standard. A calibration curve was prepared using 1,8-cineol purchased from SEIKOTEC Co., LTD(http://seikonet.jp/international) to determine the concentration. We identified the cineol contained in VOCs by a comparison of the mass spectrum with the NIST11 library.

## Results of the First Two Experiments

**Figure 3:**
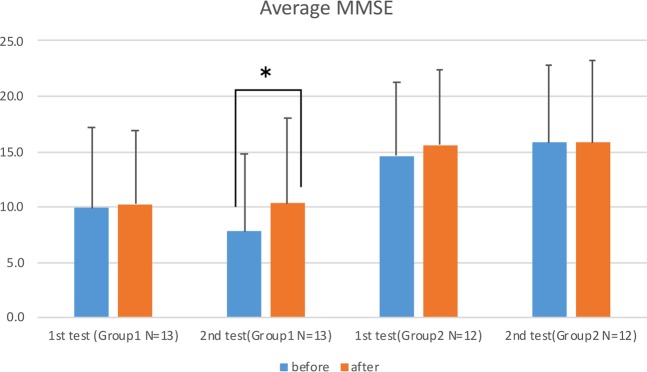
Bar graphs of average MMSE scores with standard deviation for two 1-week experiments; before (blue bar) and after (red bar). In the first experiment, 8 drops of eucalyptol oil were dispersed one hour before the subject’s wake time. In the second experiments, the same amount of eucalyptol was diffused for one hour before the subject’s wake time and also for 60 minutes at bedtime. Nonparametric tests (Wilcoxon test and Friedman test, SPSS software) were applied to determine the significant of the differences due to the small sample size of each group. Significance was established at *p* < 0.05: *and a result was considered to be trending at *p* < 0.1. *Indicates the significant improvement (p < 0.05) in the results of the second experiment with the subjects who had a diffuser in their bedrooms (Group 1).

The average level of eucalyptol in the air of the rooms with a diffuser at the pillow was 15.56 ± 1.16 μg/m^3^ in the Group Home, 17.04 ± 2.77 μg/m^3^ in Unit 1, and 20.17 ± 3.51 μg/m^3^ in Unit 2. Figure 3 shows the average MMSE for both Group 1 and Group 2 before and after the two 1-week experiments. Although the MMSE tests were performed within a relatively short period (2 weeks), we could see significant improvement in the second experiment in Group 1. It is noticeable that the MMSE of Group 1 was slightly improved during the 1^st^ test and decreased 2.4 points over the approximately 4 months between the two experiment periods, which may mean that the dementia advanced while the subjects were not exposed to the eucalyptol scent. In the second experiment, after the scent was diffused in the morning and evening in the subjects’ room for one week, the scores of Group 1 significantly improved from 7.8 to 10.3 (p = 0.002). By contrast, the average MMSE of Group 2 remained at the same level. (Fig. 3).

**Figure 4:**
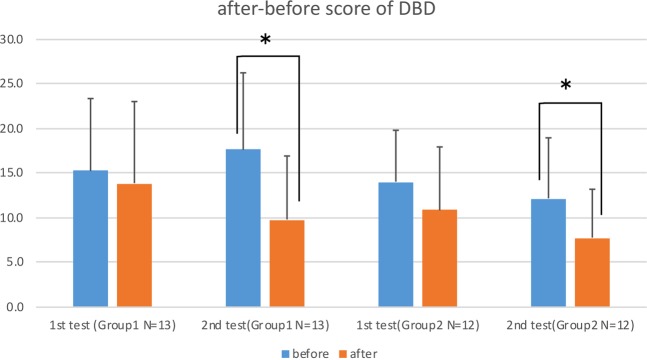
Bar graphs of after-before score of DBD with standard deviation for two 1-week experiments; before (blue bar) and after (red bar). In the first experiment, 8 drops of eucalyptol oil were dispersed one hour before the subject’s wake time. In the second experiments, the same amount of eucalyptol was diffused for one hour before the subject’s wake time and also for 60 minutes at bedtime. Nonparametric tests (Wilcoxon test and Friedman test, SPSS software) were applied to determine the significant of the differences due to the small sample size of each group. Significance was established at *p* < 0.05: *and a result was considered to be trending at *p* < 0.1. *Indicates the significant improvement (p < 0.05) in the results of the 2^nd^ experiment with both subjects who had a diffuser and who did not have a diffuser in their room (Group 1 and Group 2).

**Figure 5:**
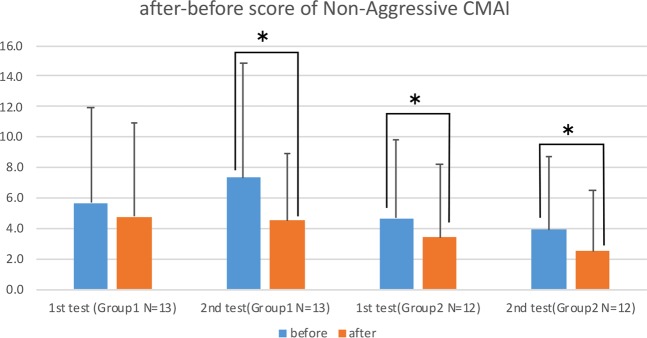
Bar graphs of after-before score of Non-Aggressive CMAI with standard deviation for two 1-week experiments; before (blue bar) and after (red bar). In the first experiment, 8 drops of eucalyptol oil were dispersed one hour before the subject’s wake time. In the second experiments, the same amount of eucalyptol was diffused for one hour before the subject’s wake time and also for 60 minutes at bedtime. Nonparametric tests (Wilcoxon test and Friedman test, SPSS software) were applied to determine the significant of the differences due to the small sample size of each group. Significance was established at *p* < 0.05: *and a result was considered to be trending at *p* < 0.1. *Indicates the significant improvement (p < 0.05) in the results of the 2^nd^ experiment with all subjects except in the first experiment with the subjects who had a diffuser in their room (Group 1).

Figures 4 and 5 show the after-before score of DBD and Non-Aggressive CMAI of each group. Higher scores on the DBD and CMAI indicate worse behavior, so a negative number means improvement in behavior after one week of the experiment. Both Group 1 and Group 2 improved their scores in both DBD and Non-Aggressive CMAI in the second experiment, and Group 2 also improved in Non-Aggressive CMAI even in the first experiment. The results of a stepwise regression for DBD and CMAI differed from the MMSE, showing that showed subjects with a higher Level of Independence had an improved DBD even without the scent being diffused in their room.

**Figure 6:**
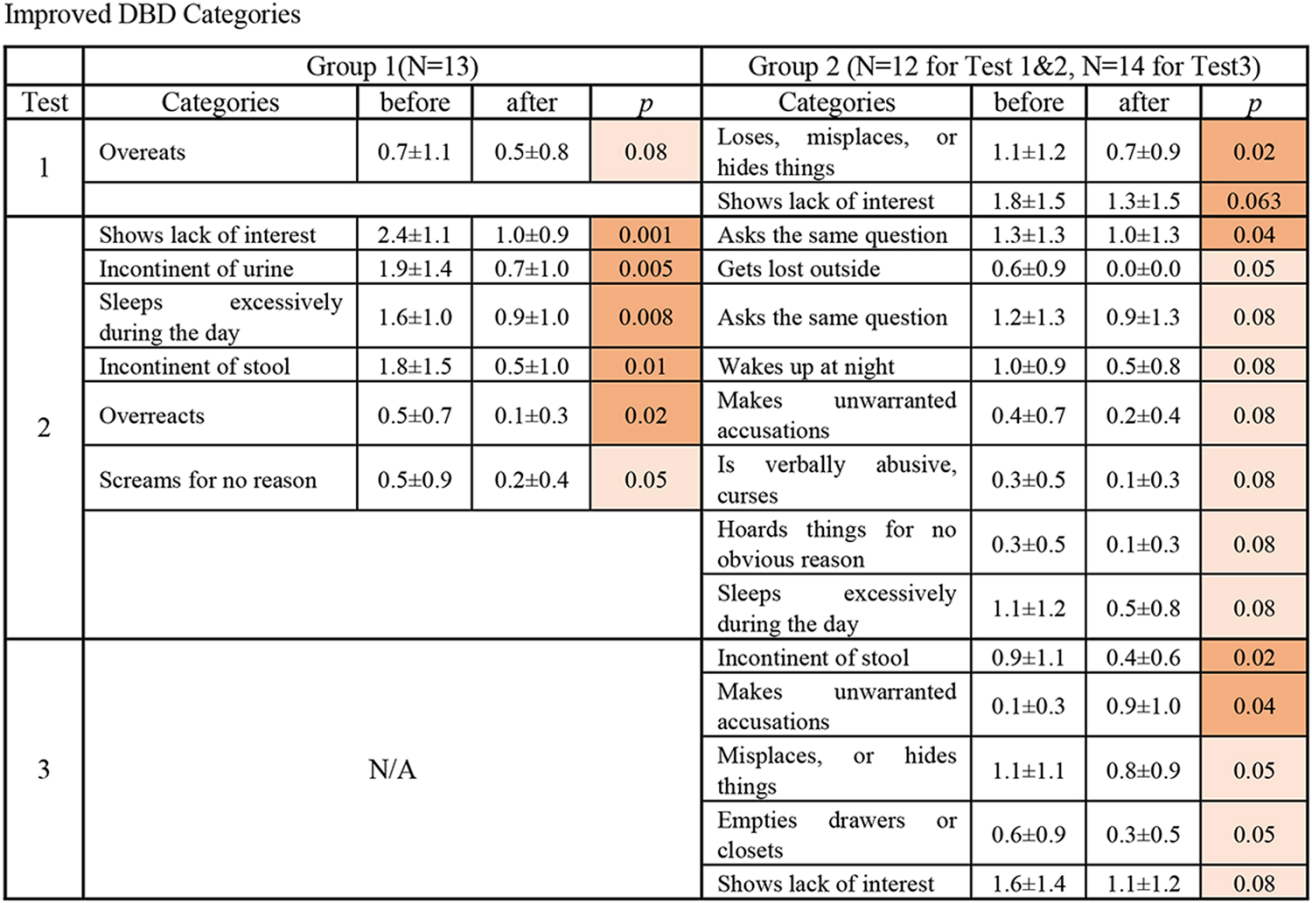
Improved categories in DBD. Nonparametric tests (Wilcoxon test and Friedman test, SPSS software) were applied to determine the significant of the differences due to the small sample size of each group. Significance was established at *p* < 0.05: *and a result was considered to be trending at *p* < 0.1. Red indicates significant improvement, and orange indicates a trend of improvement. The number of improved categories increased with both subjects who had a diffuser and who did not have a diffuser in their room (Group 1 and Group 2) in the second experiment.

**Figure 7:**
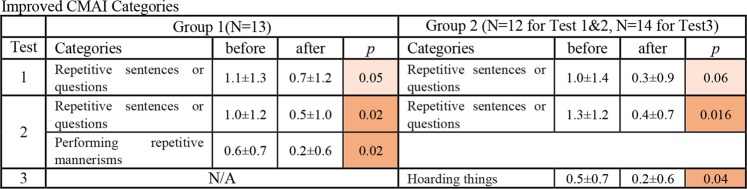
Improved categories in CMAI. Nonparametric tests (Wilcoxon test and Friedman test, SPSS software) were applied to determine the significant of the differences due to the small sample size of each group. Significance was established at *p* < 0.05: *and a result was considered to be trending at *p* < 0.1. Red indicates significant improvement, and orange indicates a trend of improvement. The number of improved categories increased with the subjects who had a diffuser in their bedroom (Group 1) and the significance of the improved category increased with the subjects who did not have a diffuser in their bedroom (Group 2) in the second experiment.

Figures 6 and 7 illustrate the changes in the DBD and CMAI categories, which showed a trend and significant change in the average score. Red indicates significant improvement, and orange indicates a trend of improvement. In the first experiment, within the categories of DBD and CMAI, Group 1 showed a trend of improvement in 1 category of DBD and 1 category of Non-Aggressive CMAI. Group 2 showed a significant improvement in 1 category and a trend of improvement in 1 category of DBD, and a trend of improvement in 1 category of Non-Aggressive CMAI. The number of improved categories increased in the second experiment. Group 1 had significant improvement in 5 categories and a trend of improvement in 1 category of DBD, and a significant improvement in 2 categories of Non-Aggressive CMAI. Group 2 showed a significant improvement in 1 category, a trend of improvement in 7 categories of DBD, and a significant improvement in 1 category of Non-Aggressive CMAI. Interestingly, Group 2, subjects who did not have a diffuser in their room, improved in multiple categories in the first and second experiment.

**Figure 8:**
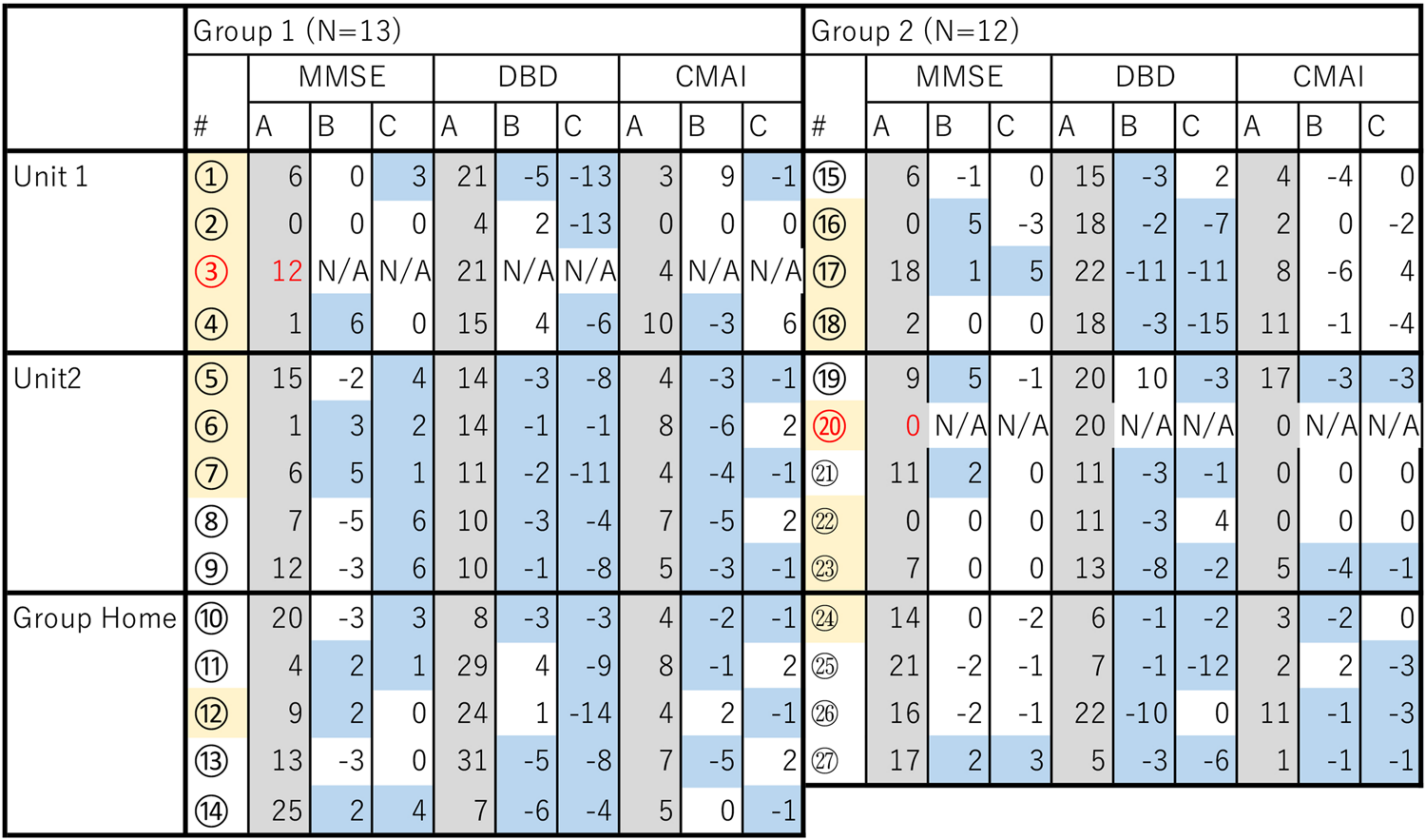
Changes in MMSE, DBD, and CMAI value of subjects before and after the two 1-week experiments. In the first experiment, 8 drops of eucalyptol oil were dispersed one hour before the subject’s wake time. In the second experiments, the same amount of eucalyptol was diffused for one hour before the subject’s wake time and also for 60 minutes at bedtime. “A” indicates the value before the first experiment, “B” indicates the change in values during the first experiment, and “C” indicates the change during the second experiment. A positive number indicates improvement in MMSE, while a negative number indicates improvement in DBD and CMAI. Blue indicates improvement, grey indicates the score before the 1^st^ experiment, orange indicates residents who did not participate in any activity, and  red indicates subjects who passed away during the experiment. All subjects of the subjects who did not have a diffuser in their room in the smallest unit (unit 2 in Group 2) improved the MMSE, DBD, or CMAI either from the 1^st^ or the 2^nd^ experiment.

Figure 8 shows the difference in MMSE, DBD, and CMAI scores before and after the two 1-week experiments for Group 1 and Group 2. “A” indicates the value before the first experiment, “B” indicates the change in values during the first experiment, and “C” indicates the change during the second experiment. A positive number indicates improvement in MMSE, while a negative number indicates improvement in DBD and CMAI. Blue indicates improvement, grey indicates the score before the 1^st^ experiment, and orange indicates residents who did not participate in any activity Red indicates subjects who passed away during the experiment. In this table, we can see that all subjects of unit 2 in Group 2 improved the MMSE, DBD, or CMAI either from the 1^st^ or the 2^nd^ experiment (Fig. 8). Follow-up tests with Group 2 of DBD and CMAI were conducted 3 weeks after the 2^nd^ test. The result did not show any improvement in scores, which confirmed that Group 2 also improved in DBD and CMAI only during the weeks when the scent was diffused.

## Results of the Third Experiment

**Figure 9:**
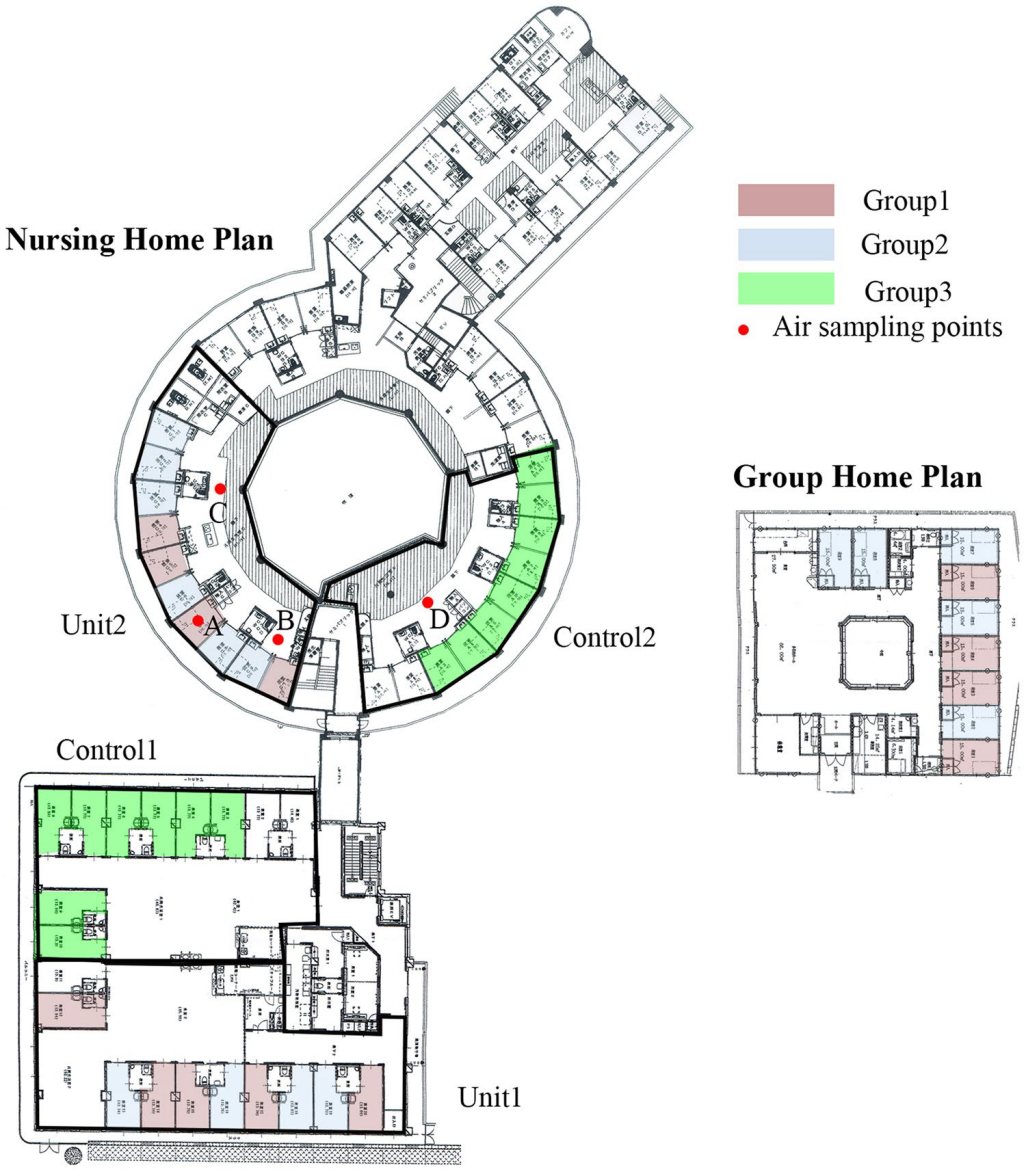
Floor Plan. Pink indicates the room with a diffuser. Blue indicates the room with no diffuser. Green indicates the room without diffuser in the unit. Red dots indicate air sampling points.

Figure 9 shows the distribution of the subjects and locations where the air sample was collected. Prior to the experiment, the air in the bedroom (point A in figure 9) was sampled (see Methods) after 15 minutes of operation of the diffuser. After the 1^st^ day of the experiment the level of eucalyptol in the living room of the unit with the diffuser was tested at 2 locations (point B and C in figure 9) and in the living room of the unit with no diffuser at one location (point D in figure 9). The resident of the room in front of point B, the subject of Group 1, woke up at 9:00 AM and the door was kept open after 9:05 AM. The air was collected at point B, C, and D at 9:15 AM and point B and C at12:15 PM. As the outside temperature was mild on this day, the air conditioner in the unit was not in operation in the morning. The analysis of the volatiles with GC/MS showed that the concentration ratio of eucalyptol in the air outside the bedroom at points A: B: C: D at 9:15 was 100:63:23.2:1.4 respectively, and at points A: B: C at 12:15 PM was 100:32.2:7.6. Thus the eucalyptol drifted into the living room, traveling as far as the next room across the corridor. As the door to the unit is generally kept closed, the eucalyptol scent remained in the living room during the morning. From this result, we surmise that residents and caregivers in Unit 1, Unit 2, and the Group Home were exposed to an level of eucalyptol that was unnoticeable to the caregivers in the living room during the one-week period of the experiment.

Compared with the result of the 2^nd^ experiment, conducted 4 months after the 1^st^ experiment with same groups of subjects, the results of the 3^rd^ experiment, conducted 13 months after the 2^nd^ experiment with a new group of subjects, were weaker. There was no significant improvement in MMSE and CMAI for all groups. This result may indicate the influence of the 1^st^ experiment on the 2^nd^. Only Group 2 showed a trend of improvement in overall DBD (*p* = 0.066) in the 3rd experiment. Within the categories of DBD and CMAI, Group 2 showed a trend of improvement in 5 categories of DBD, and a significant improvement in one category in CMAI.

**Figure 10:**
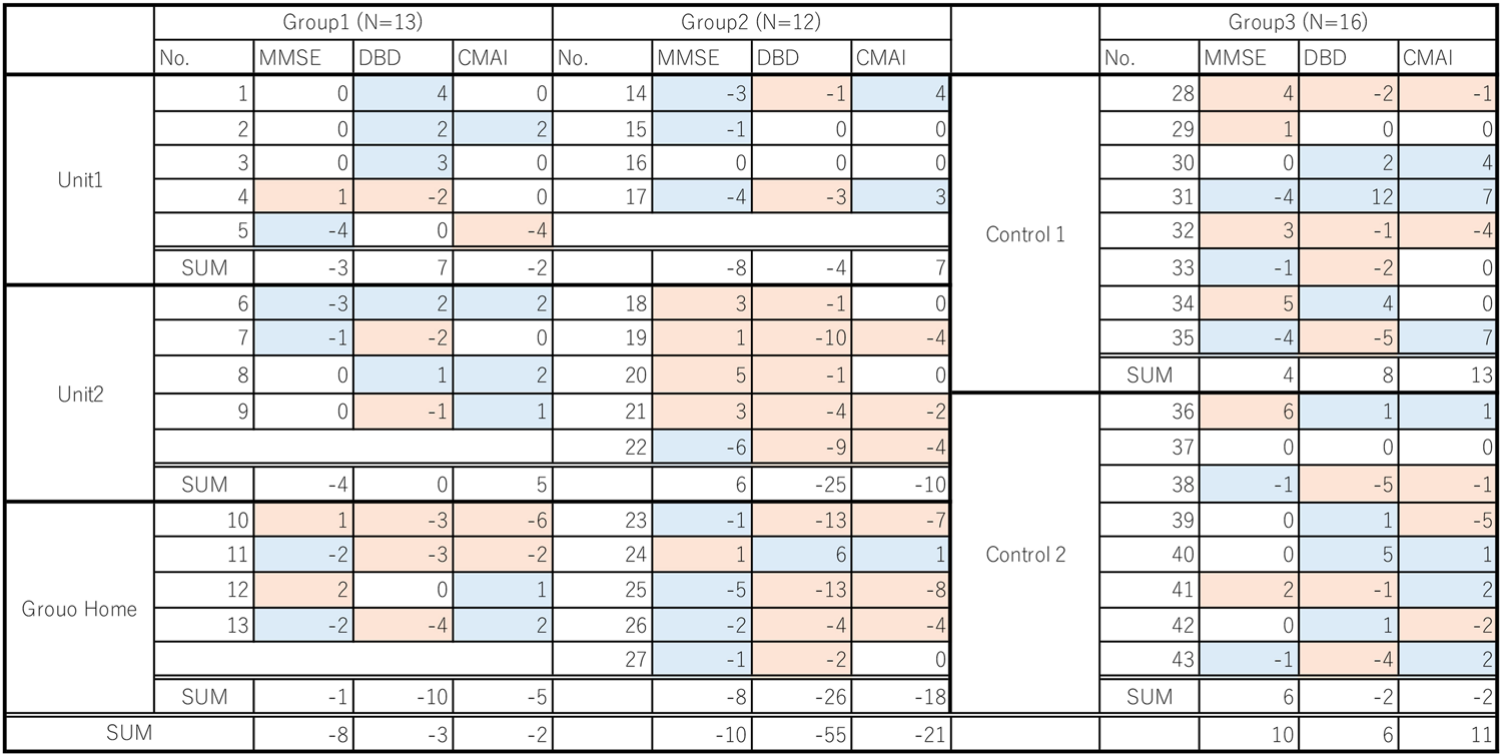
Changes in MMSE, DBD, and CMAI. Pink indicates improvement. Blue indicates deterioration. Positive numbers indicate improvement in MMSE, and negative numbers indicate improvement in DBD and CMAI. All residents in the smallest unit (unit 2), both who had a diffuser and who did not have a diffuser in their room, improved in DBD and CMAI which suggests that one-week exposure to an unperceivable level of eucalyptol improved the behavior of the subjects.

Figure 10 summarizes the change in the scores of the MMSE, DBD, and CMAI for all subjects. Pink indicates improvement and blue indicates deterioration. Positive numbers indicate improvement in MMSE, and negative numbers indicate improvement in DBD and CMAI. Although the MMSE score got worse, the scores in DBD and CMAI improved in both Group 1 and Group 2. Particularly, for the residents of unit 2, all subjects improved in DBD and CMAI, and 4 subjects out of 5 in Group 2 improved in MMSE. However, whereas MMSE scores improved, DBD and CMAI worsened in Group 3. Improvement of the DBD differed significantly between Group 2 and Group 3 (*p* = 0.041). Furthermore, within the categories of DBD, Group 3 significantly deteriorated in 1 category (Overreacts, *p* = 0.03). From this result, we can say that the one-week exposure to an unperceivable level of eucalyptol to the subjects did not improve MMSE, but improved the behavior of the subjects.

## Discussion

The results when viewed in total, indicate that the smell of a single identified chemical compound, eucalyptol, has a measurable impact on the cognitive and behavioral status of elderly persons who were cognitively impaired. The effects were not large, but this is to be expected since the instruments used, in particular the MMSE, are not as effective in the types of short term explorations reported here. Significant improvements were found in representative tests in all domains examined. This includes changes in the responses of subjects whose room never had a diffuser present (Group 2/Unit 2).

**Figure 11:**
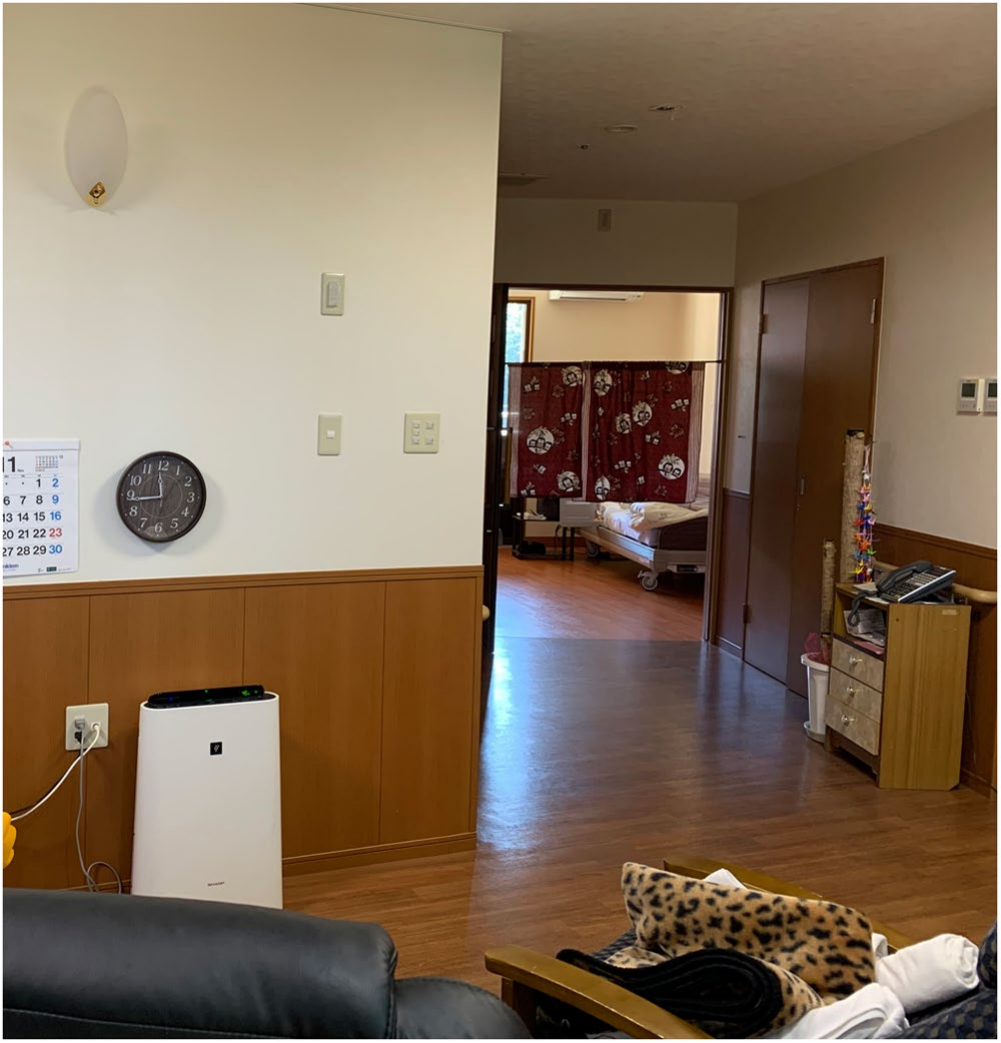
Daytime spatial relationship of living room and the private room in Unit 2.

Why did subjects in Group 2, particularly subjects in unit 2, improve their behavior during the week without having a diffuser in their bedrooms? When we look at the routine of N. Home, the caregivers call the subjects around individual wake-up time, help them change, and take them to the living room for a meal. All residents have breakfast and spend the rest of the day in the living room while the doors to their rooms are kept wide open (Fig. 11). As the diffuser was set to operate for 60 minutes before individual wake up time, the caregiver opened the doors around when the diffuser finished diffusing the scent. As figure1 shows, among 3 units, unit 2 has the smallest private rooms and common space. Perhaps some diffused scent from the subjects’ rooms entered the living room when the door was opened after the subjects woke, and both subjects and the control group experienced some effect from the scent drifting into the living room during the day.

In N. Home, because caregivers change every day, it was impossible to have questionnaires based on continuous observation of residents. However, we had anecdotes from caregivers during the experiments. For example, they commented on improvements of facial expression, better social interactions, and decreased accidents as a result of subjects becoming more collaborative. During the 2^nd^ experiment, the caregivers reported that a subject of Unit 2 with the diffuser in her room woke up at midnight and had trouble going back to sleep, which was unusual. This may indicate that the concentration of eucalyptol was too strong for her even though, when asked, she did not perceive the scent. Above all, all staff perceived that the eucalyptol scent in the subjects’ rooms masked other odors, so that staff could complete their tasks more comfortably given the fresher air.

Although the effect of aromatherapy is often linked with a preference for the aroma^25,26^, the notable finding of this experiment was that the effects of long-term exposure to a low level of eucalyptol upon the behavior of an elderly population with dementia occurred without olfactory perception. With respect to the effect of a subtle amount of volatile substance, Matsubara reported that a decline in attention during the visual discrimination task for 30 min. was significantly diminished by a low dose volatile of the leaves of *Laurus noblis L*.(laurel), though a high-dose volatile was not effective^27^. Because eucalyptol is the main component of *Laurus noblis L*., this study may also confirm the effect of the drifting scent of eucalyptol.

Currently, various programs such as exercise therapy and horticultural therapy are available as non-pharmacological therapies in many medical facilities and nursing homes. However, because these programs require the preparation of tools, materials, and instructors, they are difficult to organize in understaffed facilities. By contrast, aroma therapy can be applied in any facility because it requires minimum time for preparation and expense. The results of this pilot study indicate that eucalyptol has the potential to be an effective therapeutic option even as a low-dose odorant. It does not appear to matter whether or not a person can detect the scent. Its presence can improve the cognitive performance and mood of elderly patients. Because the effect of the scent is not associated with a personal preference for an odorant, this finding can be applied anywhere and anytime.

## Limitations

Although this study provides evidence of the effectiveness of eucalyptol for people with dementia, because the study was conducted in an actual nursing home, the size and layout of each unit and the number of times for opening and closing doors of each unit were not controllable. In addition, the number of subjects tested within one facility, the duration of the experiment, and the flexibility in the spatial setting were all very limited. The study found that the potential effectiveness of eucalyptol applies not only to person in the room with the diffuser, but also to caregivers. Our study, however, does not yet allow us to specify the appropriate dose and length of exposure that are most effective. The usage of eucalyptol at different durations and concentrations should be tested and subgroup analysis should be conducted with more subjects who have different types or severities of dementia. Furthermore, as the study focused on eucalyptol, it is not certain if the effects is observed only with eucalyptol or with the presence of other scents. There might be different volatile with similar or better effects than eucalyptol. Further research on the effects of other volatiles is needed.

## Supplementary information

Supplementary document.

Consent Form.

Consent Form 2.

## References

[1] Ali B (2015). Essential oils used in aromatherapy: A systemic review. Asian Pacific Journal of Tropical Biomedicine..

[2] Clarke, S. *Essential chemistry for aromatherapy*; 10.1016/B978-0-443-10403-9.X0001-5 (Churchill Livingstone Elsevier, 2008).

[3] Sánchez-Vidaña H, Rachel S (2009). Aromatherapy facts and fictions: A scientific analysis of olfactory effects on mood, physiology and behavior. International Journal of Neuroscience..

[4] Bonnafé E (2017). Monoterpenoid-based preparations in beehives affect learning, memory, and gene expression in the bee brain. Environmental Toxicology and Chemistry..

[5] Moss M, Hewitt S, Moss L, Wesnes K (2008). Modulation of cognitive performance and mood by aromas of peppermint and ylang-ylang. J. Neuroscience..

[6] Goto S, Puzio H, Kamal N, Herrup K (2014). Differential responses of individuals with late-stage dementia to two novel environments: A multimedia room and an interior garden. Journal of Alzheimer’s Disease..

[7] Akiyoshi K (2013). The relax effect which the degree of the likes and dislikes of the aroma gives to man. Bulletin of Nara Medical University School of Nursing..

[8] Sarid O, Zaccai M (2016). Changes in mood states are induced by smelling familiar and exotic fragrances. Front. Psychol..

[9] Sun H (2015). Identification of floral scent in chrysanthemum cultivars and wild relatives by gas chromatography-mass spectrometry. Molecules..

[10] Tisserand, R. & Balacs, T. *Essential oil safety: A guide for health care professionals*. (Churchill Livingstone Elsevier, 1995).

[11] Forrester LT (2014). Aromatherapy for dementia. Cochrane Database of Systematic Reviews..

[12] Dong L, Wang J, Deng C, Shen X (2007). Gas chromatography-mass spectrometry following pressurized hot water extraction and solid-phase micro extraction for quantification of eucalyptol, camphor, and borneol in chrysanthemum flowers. Journal of Separation Science..

[13] Yang L (2017). Analysis of floral volatile components and antioxidant activity of different varieties of *Chrysanthemum morifolium*. Molecules..

[14] Juergens LJ (2017). Anti-inflammatory effects of 1,8-cineole (eucalyptol) improve glucocorticoid effects *in vitro*: A novel approach of steroid-sparing add-on therapy for COPD and asthma?. Synergy..

[15] Kovar KA, Gropper B, Friess D, Ammon HPT (1987). Blood levels of 1,8 cineol and locomotor activity of mice after inhalation and oral administration of rosemary oil. Planta Medica..

[16] Kehrl W, Uwe S, Uwe D (2004). Therapy for acute nonpurulent rhinosinusitis with cineole: Results of a double-blind, randomized, placebo-controlled trial. Laryngoscope..

[17] Stimpfl T (1995). Concentration of 1,8 cineole in blood during prolonged inhalation. Chemical Senses..

[18] Soares M.C.M.S., Damiani C.E.N., Moreira C.M., Stefanon I., Vassallo D.V. (2005). Eucalyptol, an essential oil, reduces contractile activity in rat cardiac muscle. Brazilian Journal of Medical and Biological Research.

[19] Ambrosch S (2018). Effects of 1,8-cineole and (–)-linalool on functional brain activation in a working memory task. Flavour and Fragrance Journal..

[20] Kilonzi F (2019). A pilot study on the effects of chrysanthemum scent on memory and mood. Journal of Therapeutic Horticulture..

[21] Ministry of Health, Labour and Welfare. *Health and Welfare Services for the Elderly*, https://www.mhlw.go.jp/stf/seisakunitsuite/bunya/hukushi_kaigo/kaigo_koureisha/nintei/index.html (2019).

[22] Arevalo-Rodriguez I (2015). Mini-Mental State Examination (MMSE) for the detection of Alzheimer’s disease and other dementias in people with mild cognitive impairment (MCI). The Cochrane database of systematic reviews..

[23] Machida, Ayako (2012). DBD Dementia Behavior Disturbance Scale (DBD) :*tanshukuban no sakusei oyobi shinraisei*. Journal of the Japan Geriatrics Society..

[24] Griffiths AW, Christopher PA, Burnley NL, Creese B (2020). Validation of the Cohen-Mansfield Agitation Inventory Observational (CMAI-O) tool. International Psychogeriatrics..

[25] Holmes C (2002). Lavender oil as a treatment for agitated behavior in severe dementia. International Journal of Geriatric Psychiatry..

[26] Yamada, K. and Iwasaki, T. *Kaori no shukosei to performance*. *Japan Society for Research on Emotions*:*13th Conference Proceedings* (2005).

[27] Matsubara. E (2011). Volatiles emitted from the leaves of *Laurus nobilis L*. improve vigilance performance in visual discrimination task. Biomedical Research.

