# Supplementary material for: The Effect of Eucalyptol on Nursing Home Residents
Source: Sci Rep. 2020 Mar 4;10:3996. doi: 10.1038/s41598-020-61045-8 (PMC7055304; doi:10.1038/s41598-020-61045-8)
Supplement: Supplementary file 1 — Supplementary document. [file 41598_2020_61045_MOESM1_ESM.pdf]

The Effect of Eucalyptol on Nursing Home Residents

Seiko Goto[1,*], Hinako Suzuki[2], Toshinori Nakagawa[3], Kuniyoshi Shimizu[4]

[1] Nagasaki University, School of Environmental Science, Nagasaki, 852-8521, Japan.

[2] Nagasaki University, School of Environmental Science, Nagasaki, 852-8521, Japan.

[3] Shiga University, School of Environmental Science, Shiga, 522-8533, Japan.

[4] Kyushu University, Faculty of Agriculture, Fukuoka, 819-0395, Japan.

*gotos@nagasaki-u.ac.jp

DBD

The Dementia Behavior Disturbance Scale (DBD scale) is a questionnaire for caregivers to measure the behavioral symptoms of patients. The DBD scale is comprised of the following 28 questions about dementia-related behavior, scored by an observer on a 5-point scale (Score 0: Never; 4: Have the symptom all the time).

1. Asks the same question over and over again.
2. Loses, misplaces, or hides things.
3. Shows lack of interest in daily activities.
4. Wakes up at night for no obvious reason.
5. Makes unwarranted accusations.
6. Sleeps excessively during the day.
7. Wanders aimlessly outside or in the house during the day.
8. Repeats the same action (e.g., wiping table) over and over again.
9. Is verbally abusive, curses.
10. Dresses inappropriately.
11. Screams for no reason.
12. Refuses to be helped with personal care tasks, such as bathing, dressing, brushing teeth.
13. Hoards things for no obvious reason.
14. Moves arms or legs in a restless or agitated way.
15. Empties drawers or closets.
16. Wanders in the house at night.
17. Gets lost outside.
18. Refuses to eat.
19. Overeats.
20. Is incontinent of urine (wets himself/herself).
21. Paces up and down.
22. Makes physical attacks (hits, bites, scratches, kicks, spits.)
23. Cries or laughs inappropriately.
24. Engages in inappropriate behaviour.
25. Exposes himself/herself indecently.
26. Destroys property or clothing, breaks things.
27. Is incontinent of stool (soils himself/ herself).
28. Throws food.

CMAI

Cohen-Mansfield Agitation Inventory (CMAI) is a questionnaire for caregivers to assess agitation related to cognitive impairment. The questionnaire consists of the following 14 questions relating to aggressive behaviors and 15 relating to non-aggressive behaviors on a 7-point scale.

Physical / Aggressive

1. Hitting (including self)
2. Kicking
3. Grabbing people
4. Pushing
5. Throwing things
6. Biting
7. Scratching
8. Spitting
9. Hurting self or others
10. Tearing things or destroying property
11. Making physical sexual advances

Physical / Non-Aggressive

12. Pacing, aimless wandering
13. Inappropriate dressing or disrobing
14. Trying to get to a different place
15. Intentional falling
16. Eating / drinking inappropriate substances
17. Handling things inappropriately
18. Hiding things
19. Hoarding things
20. Performing repetitive mannerisms
21. General restlessness

Verbal / Aggressive

22. Screaming
23. Making verbal sexual advances
24. Cursing or verbal aggression

Verbal / Non-aggressive

25. Repetitive sentences or questions
26. Strange noises (weird laughter or crying)
27. Complaining
28. Negativism
29. Constant unwarranted requests for attention or help
