# Supplementary material for: The Effect of Eucalyptol on Nursing Home Residents
Source: Sci Rep. 2020 Mar 4;10:3996. doi: 10.1038/s41598-020-61045-8 (PMC7055304; doi:10.1038/s41598-020-61045-8)
Supplement: Supplementary file 2 — Consent Form. [file 41598_2020_61045_MOESM2_ESM.pdf]

The Effect of Eucalyptol on Nursing Home Residents

Seiko Goto[1,*], Hinako Suzuki[2], Toshinori Nakagawa[3], Kuniyoshi Shimizu[4]
[1] Nagasaki University, School of Environmental Science, Nagasaki, 852-8521, Japan.
[2] Nagasaki University, School of Environmental Science, Nagasaki, 852-8521, Japan.
[3] Shiga University, School of Environmental Science, Shiga, 522-8533, Japan.
[4] Kyushu University, Faculty of Agriculture, Fukuoka, 819-0395, Japan.
*gotos@nagasaki-u.ac.jp

Supporting Document #1
Consent Form (Translation)

----

1. RESERCH TITLE: The Effect of Eucalyptol on Nursing Home Residents

2. RESEARCH PERIOD: 2018 June- October

3. PURPOSE:
   This pilot study aims to analyze clinical evidence of the effect of the scent of eucalyptol on dementia patients to know whether eucalyptol aroma is an effectiveness at reducing the symptoms of dementia.

4. STUDY PROCEDURES:
   Two one-week experiments were performed. Before and after each test, the MMSE (Mini-Mental State Examination), DBD (Dementia Behavior Disturbance scale), and CMAI (Cohen-Mansfield Agitation Inventory) will be filled on the participants by the research staffs. During the second experiment, caregivers will give a simple memory test to the subject group. The scent will be diffused only at wake-up time for 60 min. in the first experiment and at wake-up time and bedtime for 60 min. in the second experiment. There is no physical test, such as wearing a monitor, drawing blood, or touching something.

5. PARTICIPATION & ALTERNATIVES TO PARTICIPATION
   Participation in this study is completely voluntary. If you decide at any point in time that you would prefer not to participate, you are welcome to stop. The consent and authorization form will not expire but you may revoke your permission at any time.

6. RISKS &DISADVANTAGE
   Confidentiality
   This research will use only an anonymous numerical ID to link minimal demographic information such as your name, address, phone number, date of birth, or any sensitive identifying information. This ID# will be entered into a spreadsheet and linked to data and we will delete all data files from computer storage devices after a 1 year.

   Risk of Aroma
   As Eucalyptol is a common component of many plants' scent, such as Eucalyptus and Rosemary, which is used in many commercial products, there are no foreseeable risks. The diffuser has the function to keep the level not get too strong. If the subject expressed discomfort, we will terminate the experiment.

7. DISSEMINTAION of RESULT
   The research result will be disseminated through the staff of the facility upon your request.

8. PUBLICATION POLICY
   The publication of the result will be taken the lead by the researchers and its copy right will be belong to the researchers of this study

9. COMPENSATION
   There is no compensation for the participation.

CONTACT

If you have questions at any time about the research or the procedures, you may contact:

Project Manager
Seiko Goto
Nagasaki University School of Environmental Science
Office Phone: 095-819-2740
E-Mail: gotos@nagasaki-u.ac.jp.

Experiment Manager
Hinako Suzuki
Nagasaki University School of Environmental Science
Phone:080-9243-9906
E-Mail: bb40115058@gmail.com

Consent and Authorization

I have read this consent form.

I have had the chance to ask questions and my questions have been answered.

I authorize the use and disclosure of information in connection with that future research, under the conditions described in this form.

Please initial your choice below:

_______ I agree to participate in this part of the research as indicated above.

OR

_______ I do not agree to participate in this part of the research as indicated above.

____
Subject Name (printed)

____ ____________________
Signature of Subject Date

____
Caregiver Name (printed)

____ ____________________
Signature of Caregiver Date

____ ____________________
Signature of Person Conducting Informed

You will receive a signed copy of this signed consent and authorization form.
