# Supplementary material for: The Effect of Eucalyptol on Nursing Home Residents
Source: Sci Rep. 2020 Mar 4;10:3996. doi: 10.1038/s41598-020-61045-8 (PMC7055304; doi:10.1038/s41598-020-61045-8)
Supplement: Supplementary file 3 — Consent Form 2. [file 41598_2020_61045_MOESM3_ESM.pdf]

The Effect of Eucalyptol on Nursing Home Residents

Seiko Goto[1.*], Hinako Suzuki[2.], Toshinori Nakagawa[3.], Kuniyoshi Shimizu[4.]

[1.] Nagasaki University, School of Environmental Science, Nagasaki, 852-8521, Japan.

[2.] Nagasaki University, School of Environmental Science, Nagasaki, 852-8521, Japan.

[3.] Shiga University, School of Environmental Science, Shiga, 522-8533, Japan.

[4.] Kyushu University, Faculty of Agriculture, Fukuoka, 819-0395, Japan.

*gotos@nagasaki-u.ac.jp

Supporting Document #2 (original consent form)
本研究にご参加いただく皆様へ

1. 研究課題名：「ユーカリプトルの香りによる効果についての研究」

2. 研究期間：平成 30 年 6 月

3. 研究目的：覚醒効果があるとされるユーカリプトルの香りが、高齢者の気分又は行動の向上に寄与するかを明らかにすることを目的とします。

4. 研究方法

　本実験では、ユーカリプトルの香りを被験者の居室に介護者が起床時間に毎日１時間、一週間薫き、被験者のアロマによる行動と気分の変化を、介護者の被験者に対する行動評価、認知能力評価、そして介護者に対するアンケートによって明らかにするものです。香りによる生理心理的効果を検討するため、器具を装着したり、身体を傷つけたり、汚したりすることはありません。

5. 研究への参加の任意性とその同意の撤回

　この研究に参加するかどうかは、あなた自身の意志で決めていただきます。また、同意された後で研究への参加を取りやめることも自由ですし、それによる不利益も全くありません。お気軽にご相談下さい。

6. 予想される危険性及び不利益

個人情報の保護

　本結果は学術目的のみに使用し、研究に参加していただく被験者の個人情報は、研究責任者及び研究分担者のみによって保管し、管理されます。また、それらのデータは、分析する前に研究分担者によって、住所・氏名・年齢などの個人を特定できる情報を削り、代わりに新しく符号をつけ連結可能匿名化され、１年間保管したのちに破棄致しますので、研究責任者及び研究分担者以外に漏洩することはありません。

アロマの危険性

　本研究で使用するユーカリプトルは、主にユーカリやローズマリーなどといった多くの植物に含まれる成分であり、一般に市販され、利用されている香料であるため、人体に影響はありません。また、使用するアロマディフューザーにおきましても、香りの濃度を調節し、一定に保つ機能があるため、身体的危険性はありません。万が一体調が悪

くなった場合は遠慮なくすぐに申し出てください。その際は、実験を中止いたします。

7. 研究成果の開示

　研究結果をあなたが望まれる場合には、施設の担当者を通じてあなたに結果を説明いたします。

8. 知的財産権について

　この研究の成果に基づいて、特許権などの知的財産権が生ずる可能性もありますが、その権利は研究者に帰属し、あなたには帰属しません。

9. 費用について

　この研究に関わる費用の負担はありません。

なお、もし実験参加のためかかった費用（交通費など）があれば、提供いたします。

□研究責任者

長崎大学大学院水産・環境科学総合研究科

五島　聖子　　　　　　　　　　印

E-mail: gotos@nagasaki-u.ac.jp

□研究実施者 1

長崎大学環境科学部環境科学科 4 年

鈴木日奈子　　　　　　　　　　印

E-mail:bb40115058@gmail.com
